# Comparison of Long-Term and Perioperative Outcomes of Robotic *Versus* Conventional Laparoscopic Gastrectomy for Gastric Cancer: A Systematic Review and Meta-Analysis of PSM and RCT Studies

**DOI:** 10.3389/fonc.2021.759509

**Published:** 2021-12-24

**Authors:** Qingbo Feng, Hexing Ma, Jie Qiu, Yan Du, Guodong Zhang, Ping Li, Kunming Wen, Ming Xie

**Affiliations:** ^1^ Department of Gastrointestinal Surgery, Affiliated Hospital of Zunyi Medical University, Zunyi, China; ^2^ Department of General Surgery, Affiliated Hospital of Yangzhou University, Yangzhou, China; ^3^ Department of Pharmacy, Affiliated Maotai Hospital of Zunyi Medical University, Zunyi, China; ^4^ Department of General Surgery, The First Clinical Medical College of Lanzhou University, Lanzhou, China; ^5^ Department of Gastrointestinal Surgery, Northern Jiangsu People’s Hospital, Yangzhou, China

**Keywords:** gastric cancer, robotic gastrectomy, laparoscopic gastrectomy, propensity score matching, meta-analysis

## Abstract

**Background:**

To investigate the perioperative and oncological outcomes of gastric cancer (GC) after robotic *versus* laparoscopic gastrectomy (RG *versus* LG), we carried out a meta-analysis of propensity score matching (PSM) studies and randomized controlled study (RCT) to compare the safety and overall effect of RG to LG for patients with GC.

**Methods:**

PubMed, Web of Science, EMBASE, and Cochrane Central Register were searched based on a defined search strategy to identify eligible PSM and RCT studies before July 2021. Data on perioperative and oncological outcomes were subjected to meta-analysis.

**Results:**

Overall, we identified 19 PSM studies and 1 RCT of RG *versus* LG, enrolling a total of 13,446 patients (6,173 and 7,273 patients underwent RG and LG, respectively). The present meta-analysis revealed nonsignificant differences in tumor size, proximal resection margin distance, distal resection margin distance, abdominal bleeding, ileus, anastomosis site leakage, duodenal stump leakage rate, conversion rate, reoperation, overall survival rate, and long-term recurrence-free survival rate between the two groups. Alternatively, comparing RG with LG, RG has a longer operative time (*p* < 0.00001), less blood loss (p <0.0001), earlier time to first flatus (*p* = 0.0003), earlier time to oral intake (*p* = 0.0001), shorter length of stay (*p* = 0.0001), less major complications (*p* = 0.0001), lower overall complications (*p* = 0.0003), more retrieved lymph nodes (*P* < 0.0001), and more cost (*p* < 0.00001).

**Conclusions:**

In terms of oncological adequacy and safety, RG is a feasible and effective treatment strategy for gastric cancer but takes more cost in comparison with LG.

## Introduction

Gastric cancer (GC) is a major public health problem and the second leading cause of cancer-related death globally (1). Gastrectomy with D2 lymphadenectomy is considered to be the standard of surgical technique for patients with GC (2). Since Kitano et al. (3) reported the first successful laparoscopic gastrectomy (LG) in 1994, LG has been routinely used for the treatment of GC worldwide. Owing to the development of the robotic surgery system, Hashizume et al. (4) performed the first robotic gastrectomy (RG) in 2003. Since then, studies on RG have been broadly reported. Although several meta-analyses have compared the safety and feasibility of RG with LG, these meta-analyses comprised a small sample size, low-quality studies, and no RCT, which limited them to deduce objective conclusions (5–7).

In 2020, the largest meta-analysis (5) which included 40 studies and 17,712 GC patients suggested that operative time and blood loss may be longer and less after RG than conventional LG. To our knowledge, although several propensity score matching (PSM) studies of RG *versus* LG have been included, no randomized controlled trial (RCT) has been included to the analysis, and most of the included studies are of low quality. In general, the gold standard to estimate the causal effects of treatments is RCT, and PSM methods can reduce selection bias and control unit balance in terms of covariates (8). In the present study, to make a more comprehensive comparison on perioperative outcomes and long-term survival after RG *versus* LG, we performed a meta-analysis, only including RCTs and PSM studies, to compare RG and LG for patients with GC.

## Methods

### Search Strategy and Study Selection

The study adhered to the PRISMA guidelines and was registered at PROSPERO with the registration number CRD42021271086 (9). Given that the first RG was reported in 2003, a systematic literature search for published PSM studies and RCT which investigated RG *versus* LG for GC was performed in PubMed, EMBASE, Web of Science, and Cochrane Central Register from January 1, 2003 to July 25, 2021 by two authors (QF and JQ). Combinations of the following keywords were used: gastric cancer, laparoscopic gastrectomy, robotic gastrectomy, propensity score matching, and minimally invasive surgery. In order to gain additional studies, the references of eligible studies were manually searched.

### Inclusion and Exclusion Criteria

All titles and abstracts were screened, and eligible studies were independently identified according to the criteria by two investigators (QF and JQ).

The inclusion criteria were as follows: (1) participants: the mean age of the patients with gastric cancer > 18, (2) types of interventions: RG and LG, (3) types of studies: PSM studies, and RCT, (4) data available on interested perioperative and oncological outcomes, and (5) studies published in English.

The exclusion criteria were as follows: (1) studies including non-gastric cancer patients and (2) editorials, abstracts, letters, case reports, and expert opinion and non-English studies.

### Data Extraction and Quality Assessment

The original data from all candidate articles were individually assessed and extracted by two reviewers (QF and JQ) by using a unified datasheet, and any ambiguity was resolved by a third researcher (HM). The major data extracted include the following: name of first or corresponding author, study design, publication year, country, sample size, mean age, gender, body mass index, operative times, bleeding, overall complications, major complications, abdominal bleeding, ileus, anastomosis site leakage, duodenal stump leakage rate, conversion rate, reoperation, tumor size, number of retrieved lymph nodes, time to first flatus, time to oral intake, length of stay, proximal and distal margin distance, OS, and RFS. The Newcastle–Ottawa Scale (NOS) was adopted to assess the quality of the eligible PSM studies (10) and the modified Jadad scale for RCT studies (11). Every included study was independently evaluated by two authors (JQ and PL), and NOS score ≥ 6 or Jadad score ≥4 is considered as being of high quality.

### Statistical Analysis

The Review Manager 5.3 software was used for statistical analyses. The 95% confidence interval (CI) and mean difference (MD) were used for continuous data, while odds ratio (OR) was used for dichotomous data. For overall survival data, we use Engauge Digitizer v.4.1 software to extract OS and RFS data from survival curves (12). The method originally described by Hozo et al. was used to convert medians with ranges into means with standard deviations (13). Begg’s funnel plot and Egger’s test were used to assess potential publication bias. Statistical heterogeneity was quantified using Higgin’s *I* ^2^ index. A fixed-effects model (FEM) was adopted when the heterogeneity is low or moderate (*I* ^2^ <50%), while if the heterogeneity is high (*I* ^2^ ≥50%), a random-effects model (REM) was used.

## Results

### Characteristics of the Included Studies

**Figure 1:**
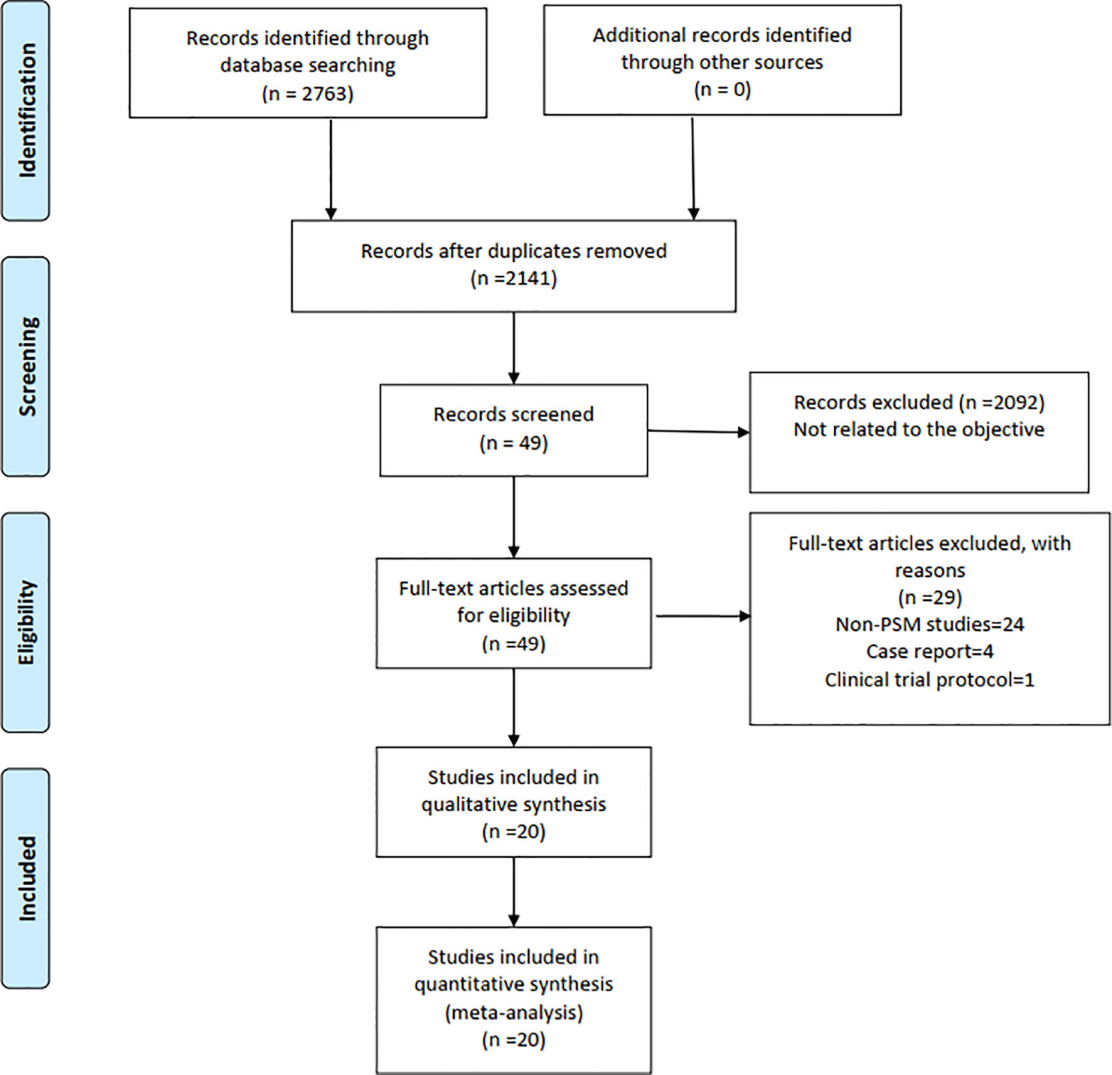
Flow chart of study identification and selection.

Finally, a total of 2,763 relevant English publications from various electronic databases was yielded. According to the inclusion criteria, 19 PSM studies (14–32) and 1 RCT (33) comparing RG and LG in a total of 13,446 patients (6,173 and 7,273 patients underwent RG and LG, respectively) were included for further analysis. A flow diagram of our analysis protocol is shown in **Figure 1**. The general information and summary of NOS scores and Jadad scores of all the included studies are given in **Table 1**. All results of interest outcomes of this meta-analysis are listed in **Table 2**.

**Table 1:** The main characteristics and NOS and Jadad scores of the included studies in this meta-analysis.

Author-year	Type of study	Period	Country	Patients (RG *vs* LG)		Age (years)		Gender (M/F)		BMI		NOS/Jadad
				RG	LG	RG	LG	RG	LG	RG	LG	
Han (14)	PSM	2008–2013	Korea	68	68	50.6 ± 8.3	49.8 ± 11.5	31/37	32/36	22.7 ± 2.4	22.8 ± 3.0	7
Kim (15)	PSM	2011–2012	Korea	185	185	53.3 ± 11.4	56.0 ± 11.5	113/72	113/72	23.8 ± 3.0	23.6 ± 2.7	8
Hong (16)	PSM	2008–2015	Korea	232	232	53.7 ± 11.5	55.0 ± 13.0	154/78	156/76	23.8 ± 3.3	23.8 ± 3.0	9
Obama (17)	PSM	2005–2009	Korea	311	311	54.5 ± 12.6	54.8 ± 12.0	187/124	186/125	23.6 ± 3.1	23.2 ± 2.8	9
Parisi (18)	PSM	2014–2015	Italy	151	151	68.81 ± 12.12	65.82 ± 14.16	70/81	66/85	24.58 ± 3.0	24.02 ± 2.22	8
Gao (19)	PSM	2011–2014	China	163	163	60.27 ± 10.50	59.88 ± 11.72	121/42	125/38	23.77 ± 3.11	23.25 ± 3.26	8
Lu (20)	PSM	2016–2017	China	101	303	NA	NA	73/28	212/91	NA	NA	6
Zhao (21)	PSM	2013–2017	China	112	112	55.6 ± 11.3	56.1 ± 11.1	78/34	79/33	23.6 ± 2.9	23.6 ± 3.0	8
Wang (22)	PSM	2016–2018	China	223	223	57.7 ± 10.9	57.4 ± 11.1	183/40	180/43	22.1 ± 3.5	22.2 ± 3.4	7
Kong (23)	PSM	2014–2017	China	266	532	58.68 ± 10.54	58.92 ± 9.82	197/69	383/149	24.23 ± 3.06	24.25 ± 3.34	8
Li 2020 (24)	PSM	2010–2019	China	516	516	55.10 ± 10.24	54.63 ± 11.85	354/162	333/183	NA	NA	8
Yang (25)	PSM	2010–2017	China	126	126	60.33 ± 8.94	60.78 ± 9.05	105/21	100/26	22.10 ± 2.48	22.13 ± 2.84	8
Ye (26)	PSM	2014–2019	China	285	285	57.1 ± 8.3	57.0 ± 8.6	189/96	186/99	24.4 ± 2.3	24.5 ± 2.2	8
Ryan (27)	PSM	2010–2014	USA	631	1262	64.5 ± 11.9	65.1 ± 11.8	449/182	906/356	NA	NA	8
Shibasaki (28)	PSM	2009–2019	Japan	354	354	67 (30–89)	66 (24–90)	230/124	230/124	22.8 (14.3–32.0)	22.4 (14.9–37.3)	9
Isobe (29)	PSM	2018–2020	Japan	50	50	69.2 ± 1.4	69.3 ± 1.4	31/19	34/16	23.0 ± 3.6	22.9 ± 2.7	8
Roh (30)	PSM	2009–2018	Korea	74	74	53.8 ± 11.6	54.6 ± 12.7	42/32	42/32	23.6 ± 2.9	23.8 ± 3.4	7
Li 2021 (31)	PSM	2010–2017	China	408	408	56.8 ± 10.0	56.1 ± 10.9	300/108	286/122	21.80 ± 2.82	21.66 ± 2.94	7
Zhou (32)	PSM	2010–2019	China	1,776	1,776	57.6 ± 10.9	57.8 ± 10.9	1,276/500	1,279/497	22.5 ± 3.2	22.4 ± 3.2	9
Lu (33)	RCT	2017–2020	China	141	142	59.4 ± 10.2	59.3 ± 11.3	94/47	90/52	23.2 ± 3.0	22.7 ± 3.3	4

RG, robotic gastrectomy; LG, laparoscopic gastrectomy; M/F, male/female; PSM, propensity-score matching; RCT, randomized controlled trial, NOS, Newcastle–Ottawa Scale; NA, not available.

**Table 2:** Summary results of the meta-analyses.

Outcomes of interest	Studies, *n*	RG	LG	OR (95%CI)	*P*-value	Heterogeneity			
						*χ* ^2^	df	*I* ^2^, %	*P*-value
Short-term outcomes									
Operative time (min)	17	4,993	5,461	39.97 (31.15, 48.79)	<0.00001	415.92	16	96	<0.00001
Blood loss (ml)	16	4,925	5,393	-15.87 (-23.35, -8.39)	<0.0001	63.05	15	76	<0.00001
Proximal resection margin (cm)	6	1,117	1,319	-0.02 (-0.20, 0.17)	0.85	4.75	5	0	0.45
Distal resection margin (cm)	6	1,117	1,319	0.07 (-0.13, 0.27)	0.51	5.71	5	12	0.34
Number of retrieved lymph node	16	5,004	5,837	1.75 (0.90, 2.60)	<0.0001	50	15	70	<0.0001
Overall complications	17	4,823	5,292	0.81 (0.72, 0.91)	0.0003	22.65	16	29	0.12
Major complications	17	4,780	5,249	0.67 (0.55, 0.82)	0.0001	15.07	16	0	0.52
Conversion rate	4	2,301	2,567	0.66 (0.40, 1.07)	0.09	1.76	3	0	0.62
Anastomosis site leakage	14	4,474	4,943	0.93 (0.67, 1.29)	0.67	10.27	13	0	0.67
Ileus	12	4,424	4,893	0.82 (0.52, 1.28)	0.38	7.69	11	0	0.74
Abdominal bleeding	12	4,001	4,470	0.66 (0.41, 1.07)	0.09	6.60	11	0	0.83
Anastomotic stenosis	5	2,630	2,630	1.00 (0.48, 2.08)	1.00	2.92	4	0	0.57
Duodenal stump leakage	6	3,192	3,458	0.88 (0.53, 1.45)	0.61	2.93	5	0	0.69
Reoperation	5	1,197	1,464	0.63 (0.33, 1.20)	0.16	1.21	4	0	0.95
Time to first flatus (day)	12	4,270	4,538	-0.14 (-0.22, -0.07)	0.0003	31.32	11	65	0.001
Time to oral intake (day)	15	4,604	5,673	-0.12 (-0.18, -0.06)	0.0001	17.53	14	20	0.23
Length of stay (day)	18	5,765	6,562	-0.31 (-0.47, -0.15)	0.0001	22.58	17	25	0.16
Cost (USD)	4	2,255	2,723	0.34 (0.32, 0.36)	<0.00001	3.32	3	10	0.34
Oncological outcomes									
OS	7	3,475	4,106	0.96 (0.86, 1.07)	0.50	3.06	6	0	0.80
RFS	5	2,732	2,732	0.98 (0.80, 1.21)	0.85	2.29	4	0	0.68

RG, robotic gastrectomy; LG, laparoscopic gastrectomy; MD, mean difference; OR, odds ratio; CI, confidence interval.

### Short-Term Outcomes

#### Operative Time

**Figure 2:**
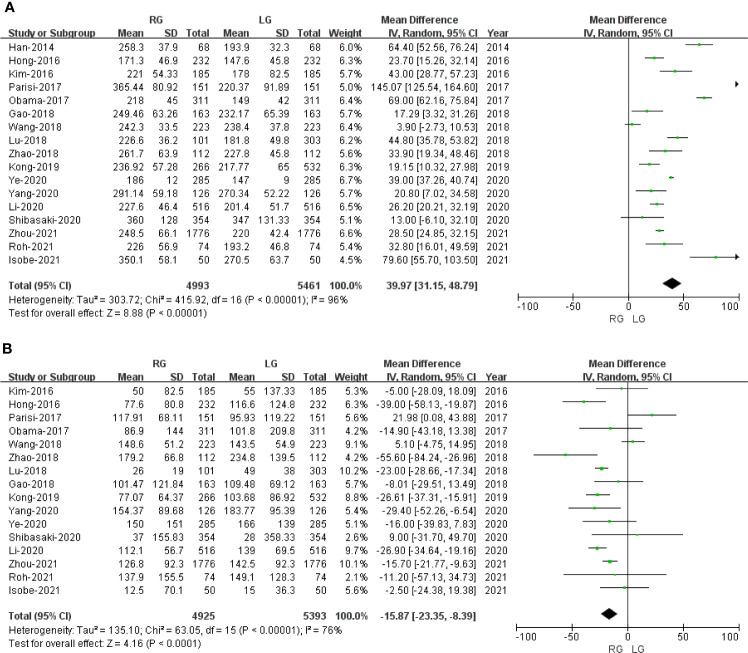
Forest plot depicting the short-term outcomes of robotic gastrectomy *versus* laparoscopic gastrectomy. **(A)** Operative time. **(B)** Blood loss.

Seventeen of the included 20 studies that encompassed 10,454 patients (4,993 and 5461 underwent RG and LG, respectively) reported operative time. The present meta-analysis showed that operative time was longer in the RG group (MD: 39.97 min; 95% CI: 31.15 to 48.79; *p* < 0.00001). Heterogeneity was high (*I* ^2^ = 96%) and analyzed in REM (**Figure 2A**).

#### Blood Loss

Sixteen studies with a total of 10,318 patients had reported blood loss. A meta-analysis of 16 studies indicated that RG had lesser blood loss compared to LG (MD: -15.87 ml; 95% CI: -23.35 to -8.39; *p* < 0.0001). Heterogeneity was high (*I* ^2^ = 76%) and analyzed in REM (**Figure 2B**).

#### Proximal Resection Margin

**Figure 3:**
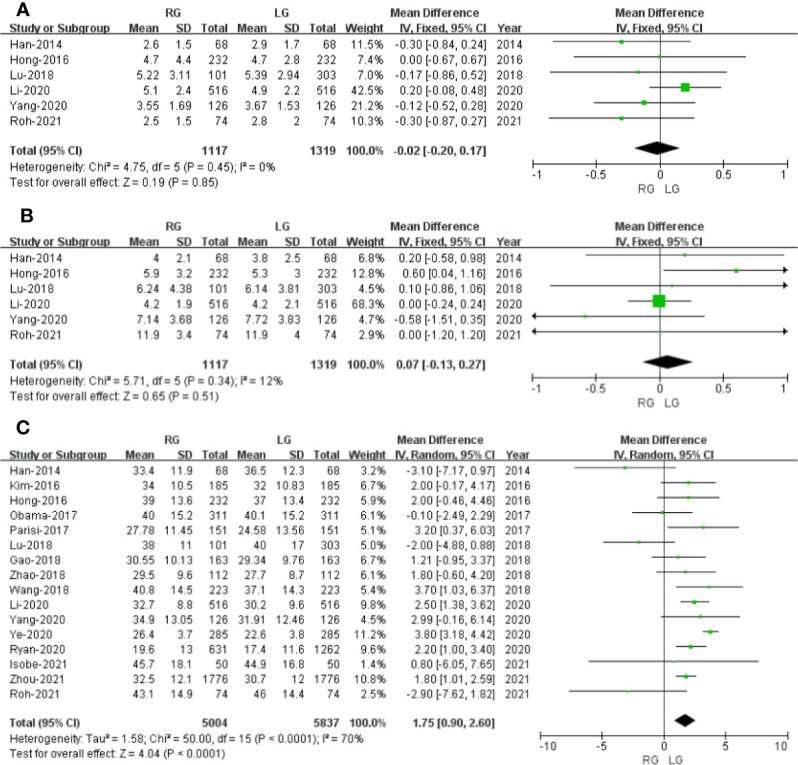
Forest plot of the comparison of robotic gastrectomy *versus* laparoscopic gastrectomy. **(A)** Proximal resection margin distance. **(B)** Distal resection margin distance. **(C)** Number of retrieved lymph nodes.

Six studies with a total of 2,436 patients had reported the proximal resection margin distance. No significant difference was found between the RG and LG groups on proximal resection margin distance (OR: -0.02; 95% CI: -0.20 to 0.17; *P* = 0.85). Heterogeneity was low (*I* ^2^ = 0%) and analyzed in FEM (**Figure 3A**).

#### Distal Resection Margin

Six studies with a total of 2,436 patients had reported the distal resection margin distance. The meta-analysis suggested no difference in distal resection margin distance between the RG and LG groups (OR: 0.07; 95% CI: -0.13 to 0.27; *P* = 0.51). Heterogeneity was low (*I* ^2^ = 12%) and analyzed in FEM (**Figure 3B**).

#### Number of Retrieved Lymph Nodes

The number of retrieved lymph node data was available in 16 studies. The meta-analysis suggested that the RG group has more retrieved lymph nodes than the LG group (OR: 1.75; 95% CI: 0.90 to 2.60; *P <*0.0001). Heterogeneity was high (*I* ^2^ = 70%) and analyzed in REM (**Figure 3C**).

### Postoperative Outcomes

#### Overall Complications

**Figure 4:**
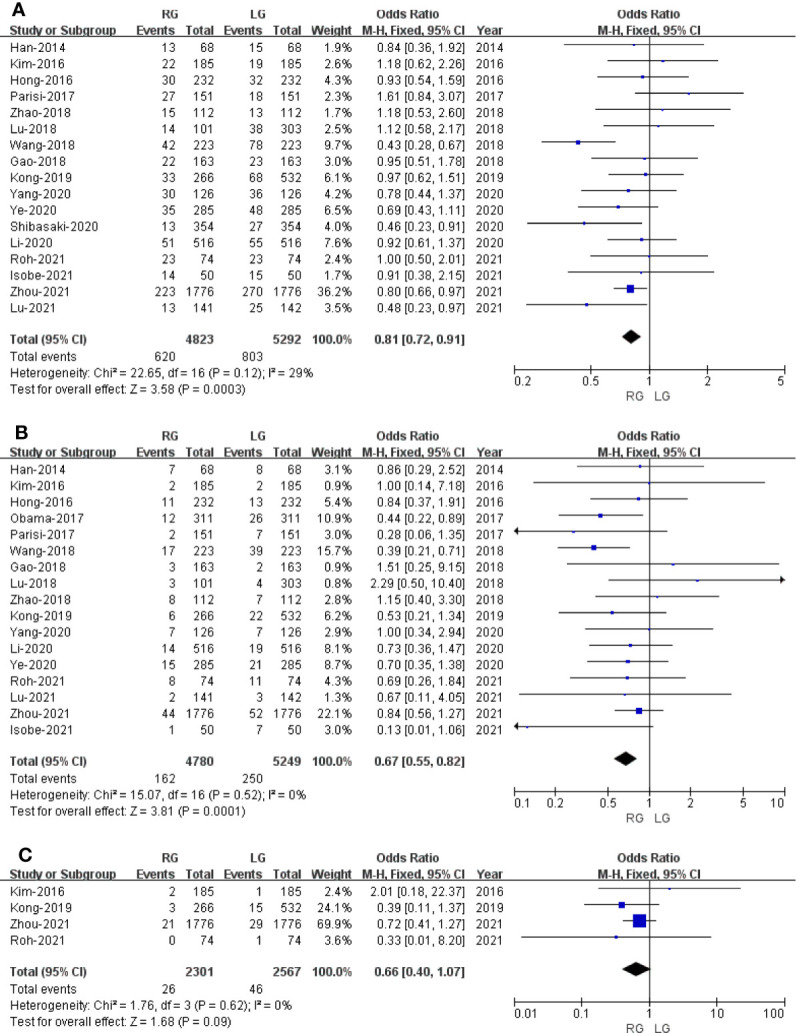
Forest plot of the comparison of robotic gastrectomy *versus* laparoscopic gastrectomy. **(A)** Overall complications. **(B)** Major complications. **(C)** Conversion rate.

Seventeen studies that encompassed 10,115 patients (4,823 and 5,292 underwent RG and LG, respectively) reported the overall complications. Data analysis of the 10,115 patients revealed lower overall complications in the RG group (OR: 0.81; 95% CI: 0.72 to 0.91; *p* = 0.0003) with low heterogeneity (*I* ^2^ = 29%), and these were analyzed in FEM (**Figure 4A**).

#### Major Complications

Major complications were based on Clavien Diendo classification; Clavien–Dindo grade ≥III was defined as major complications. Seventeen studies with a total of 10,029 GC patients reported major complications. The meta-analysis showed that the RG group presents markedly lesser major complications compared with the LG group (OR = 0.67; 95% CI: 0.55 to 0.82; *p* = 0.0001) with low heterogeneity (*I* ^2 =^ 0%), and these were analyzed in FEM (**Figure 4B**).

#### Conversion Rate

Four studies with a total of 4,868 GC patients reported a conversion rate. The meta-analysis showed that GC treated with RG presented a conversion rate similar to that of the LG group (OR = 0.66; 95% CI: 0.40 to 1.07; *p* = 0.09) with low heterogeneity (*I* ^2^ = 0%), and these were analyzed in FEM (**Figure 4C**).

#### Anastomosis Site Leakage

Fourteen studies with a total of 9,417 GC patients reported anastomosis site leakage. The difference between the rate of anastomosis site leakage was not statistically significant in the RG and LG groups (OR = 0.93; 95% CI: 0.67 to 1.29; *p* = 0.67) with low heterogeneity (*I* ^2^ = 0%), and these were analyzed in FEM.

#### Ileus

Twelve studies reported ileus. The meta-analysis indicated that the ileus rate is comparable between the RG and LG groups (OR = 0.82; 95% CI: 0.52 to 1.28; *p* = 0.38) with low heterogeneity (*I* ^2^ = 0%), and these were analyzed in FEM.

#### Abdominal Bleeding

Twelve studies with a total of 8,471 GC patients reported abdominal bleeding. The meta-analysis showed no difference in the RG and LG groups (OR = 0.66; 95% CI: 0.41 to 1.07; *p* = 0.09) with low heterogeneity (*I* ^2^ = 0%), and these were analyzed in FEM.

#### Anastomotic Stenosis

Five studies with a total of 5,260 GC patients reported anastomotic stenosis. The meta-analysis showed no difference in the RG and LG groups (OR = 1.00; 95% CI: 0.48 to 2.08; *p* = 1.00) with low heterogeneity (*I* ^2^ = 0%), and these were analyzed in FEM.

#### Duodenal Stump Leakage

Six studies with a total of 6,650 GC patients reported duodenal stump leakage. The meta-analysis suggested that RG had a duodenal stump leakage rate similar to that of the LG group (OR = 0.88; 95% CI: 0.53 to 1.45; *p* = 0.61) with low heterogeneity (*I* ^2^ = 0%), and these were analyzed in FEM.

#### Reoperation

Reoperation data was available in 5 studies. The meta-analysis indicated no significant difference between the RG and LG groups (OR = 0.63; 95% CI: 0.33 to 1.20; *p* = 0.16) with low heterogeneity (*I* ^2^ = 0%), and these were analyzed in FEM.

#### Time to First Flatus

**Figure 5:**
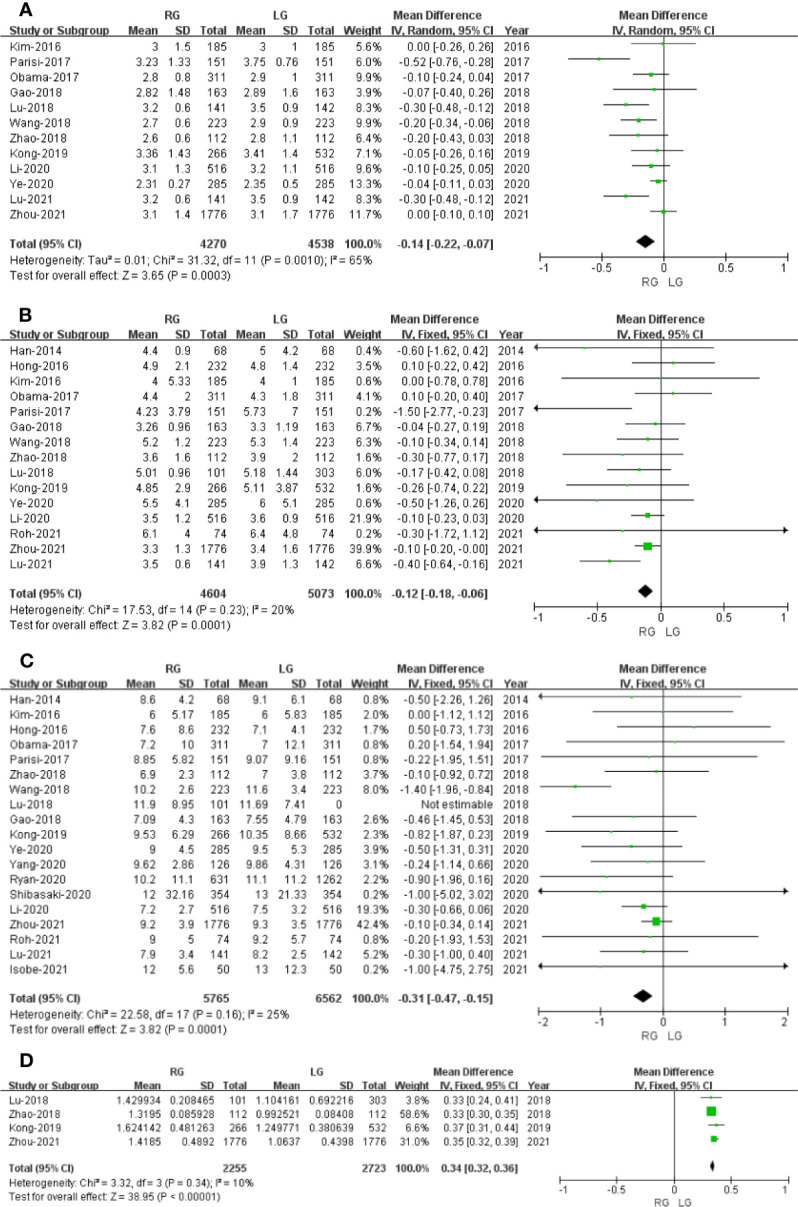
Forest plot of the comparison of robotic gastrectomy *versus* laparoscopic gastrectomy. **(A)** Time to first flatus. **(B)** Time to oral intake. **(C)** Length of stay. **(D)** Cost.

Time to first flatus data was available in 12 studies. The meta-analysis showed that the RG group has earlier time to first flatus than the LG group (OR = -0.14; 95% CI: -0.22 to -0.07; *p* = 0.0003) with high heterogeneity (*I* ^2^ = 65%), and these were analyzed in REM (**Figure 5A**).

#### Time to Oral Intake

Time to oral intake data was available in15 studies. The meta-analysis showed that the RG group has earlier time to oral intake than the LG group (OR = -0.12; 95% CI: -0.18 to -0.06; *p* = 0.0001) with low heterogeneity (*I* ^2^ = 20%), and these were analyzed in FEM (**Figure 5B**).

#### Length of Stay

Length of stay data was available in 18 studies. Noticeably, the meta-analysis showed that GC cases treated with RG presented a shorter hospital stay compared with the LG group (MD = - 0.31; 95% CI: – 0.47 to – 0.15; *p* = 0.0001) with low heterogeneity (*I* ^2^ = 25%), and these were analyzed in FEM (**Figure 5C**).

#### Cost

Four studies that included 4,978 patients (with 2,255 who underwent RG and 2,723 who underwent LG) assessed the cost. The result of meta-analysis revealed that the RG group has more cost than the LG group (OR: 0.34; 95% CI: 0.32 to 0.36; *p* < 0.00001) with low heterogeneity (*I* ^2^ = 10%), and these were analyzed in FEM (**Figure 5D**).

### Oncological Outcomes

#### Overall Survival

**Figure 6:**
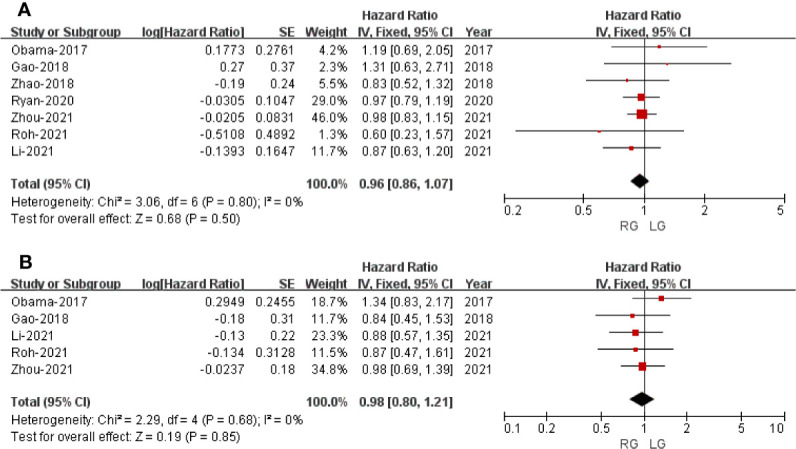
Forest plot of the comparison of robotic gastrectomy *versus* laparoscopic gastrectomy for long-term outcomes. **(A)** Overall survival. **(B)** Recurrence-free survival.

Only seven studies assessed overall survival outcome. The result of meta-analysis revealed no difference between the two groups (HR: 0.96; 95% CI: 0.86 to 1.07; *p* = 0.50) with no heterogeneity (*I* ^2^ = 0%), and these were analyzed in FEM (**Figure 6A**).

#### Recurrence-Free Survival

Only five studies that included 5,464 patients assessed recurrence-free survival outcome. The result of meta-analysis revealed no difference between the two groups (HR: 0.98; 95% CI: 0.80 to 1.21; *p* = 0.85) with no heterogeneity (*I* ^2^ = 0%), and these were analyzed in FEM (**Figure 6B**).

#### Publication Bias

**Figure 7:**
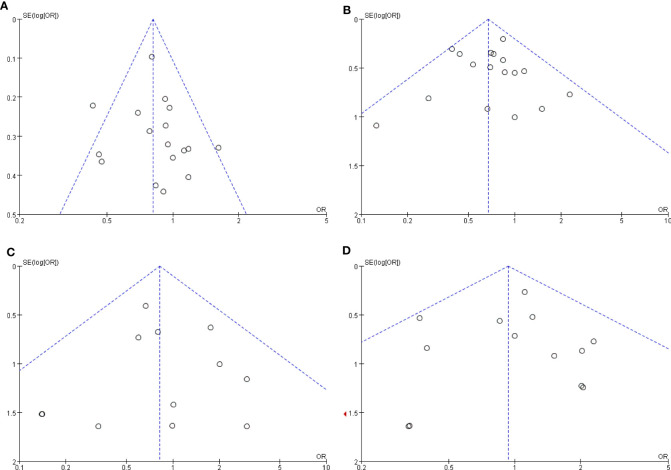
Funnel plots of the comparison of robotic gastrectomy *versus* laparoscopic gastrectomy. **(A)** Overall complications. **(B)** Major complications. **(C)** Anastomosis site leakage. **(D)** Ileus.

The publication bias was investigated by Begg’s funnel plot. All studies lie inside the 95% CIs in the funnel plot of overall complications, major complications, anastomosis site leakage, and ileus, which indicated no potential publication bias (**Figure 7**).

## Discussion

Since Hashizume et al first performed RG surgery in 2003 (4), the subsequent development of robotic equipment and the accumulation of surgical experience make RG in GC an approach on the rise, and it has now gradually become a mature surgical technique. At present, more and more studies have explored the safety and effectiveness of RG in the treatment of GC. Several studies have indicated that the treatment of gastric cancer with RG is safe and feasible, and the curative effect is similar to that of open gastrectomy, but it requires higher qualifications for the operator and is relatively time-consuming (34). At present, there are some controversies about the safety and efficacy of RG in the treatment of GC compared with LG. In order to explore the real efficacy of RG in the treatment of GC, this meta-analysis included relevant studies from 2014 to 2021 to explore the safety and effectiveness of RG and LG in the treatment of GC. All of the included studies were PSM studies, except for 1 RCT, and all of them relatively have high quality according to NOS or Jadad, as shown in **Table 1**.

Three previous meta-analyses comparing the perioperative and oncologic outcomes of RG to LG were published (5, 6, 35). However, the sample size of two meta-analyses was relatively small (6, 35), and the study of Solaini et al. (35) is only focused on RG for the treatment of GC in western patients. The study of Ma et al. (6) only focused on perioperative outcomes between RG and LG, and 19 articles with 7,275 patients were included in the study of Ma et al. The meta-analysis of Solaini et al. covered 2,034 GC participants from 10 retrospective studies. They found that RG has similar morbidity and mortality rates, less bleeding volume, and longer operative time in comparison with LG. Only the study of Ma et al. has accessed the overall survival and recurrence-free survival outcome of RG. Compared with their results, our study included some recent studies (27–33). Noticeably, all the studies are PSM studies, which can minimize selection bias. The present meta-analysis showed that RG has a longer operative time and less bleeding volume compared to LG, which was consistent with the studies of Solaini et al. and Ma et al. What is more, our study revealed that RG has a similar overall survival and recurrence-free survival outcome compared with LG for the treatment of GC and is consistent with the study of Ma et al.

Operating time is one of the most considered surgical variables when robotic surgery is compared with laparoscopy. This meta-analysis revealed that RG has a longer operative time than laparoscopy, mainly because the robotic system needs more time to set and dock. The research shows that it took about half an hour to prepare for robotic surgery (36). The meta-analysis showed that RG was associated with less blood loss. The main reason is that, with the intrinsic advantages of the robotic surgery system, surgeons can better control the bleeding of small blood vessels and reduce blood loss.

Time to first flatus and time to first oral intake are two main potential factors and play a vital role in postoperative recovery. Our meta-analysis suggested that RG had an earlier time to first flatus and oral intake. In virtue of the magnified 3D view and stable movements, RG can avoid accidental blood vessel damage and too much traction on the intestine tissues (37). With regard to complications, our study demonstrated that RG is with less overall and major complications, but there is no significant difference in terms of abdominal bleeding, duodenal stump leakage rate, conversion rate, reoperation, ileus, and anastomosis site leakage.

In regard to the proximal and distal margin and the tumor size, our study revealed that there were no significant differences in proximal and distal margin and tumor size when RG was compared to laparoscopy. Regarding the number of lymph node dissection, this meta-analysis showed that RG had more harvested lymph nodes than LG (P < 0.0001), which was contrary to the study of Guerrini et al. (5). It could be explained that RG has a magnified 3-D view and a tremor filter, which contribute to precise dissection and lymphadenectomy.

When it comes to long-term survival, to the best of our knowledge, there is still no RCT comparing the long-term survival between RG to LG in patients with GC. The largest overall survival outcome data of RG and LG in the treatment of GC comes from USA (38). Hendriksen et al. utilized the US National Cancer Data Base data which reported 4,317 patients with GC who underwent RG or LG (664 underwent RG and 3,653 underwent LG) and revealed that RG had a higher unadjusted 5-year overall survival rate than LG (50.8 and 58.9% in RG, P = 0.002), but a comparison of PSM cohorts did not show any significant difference in survival (P = 0.2611) (38). A multicenter cohort study coming from China included 5,402 GC patients. The survival data after PSM of 3,552 patients with GC (1,776 underwent RG and 1,776 underwent LG) suggested that RG and LG can achieve a 5-year overall survival rate of 80.8 and 79.5% years, respectively (p = 0.213), and RG has a 5-year disease-free survival rate similar to that of LG (79.8 vs.78.5%, p = 0.205) (32).

Overall survival (OS) and recurrence-free survival (RFS) are the most two important concerns of malignant tumor. Our meta-analysis revealed that the RG and LG groups have similar OS [HR: 0.96; 95% CI: 0.86 to 1.07; *p* = 0.50, with no heterogeneity (*I* ^2^ = 0%)] and RFS [HR: 0.98; 95% CI: 0.80 to 1.21; *p* = 0.85, with no heterogeneity (*I* ^2^ = 0%)]. Our meta-analysis revealed that RG appears to be equivalent in OS and RFS compared to LG. In some ways, the pooled data demonstrated that RG is not inferior to LG and can even achieve a superior perioperative outcome compared to LG.

Although the present meta-analysis included 19 PSM studies and 1 RCT to draw a more convincing conclusion, there are some limitations in this study to address. First, only a few studies reported long-term survival. Furthermore, high-quality RCTs with survival outcomes are expected to assess the safety and efficiency of RG for patients with GC. Additionally, only a few studies described HRs and SDs directly. For the others, we used Engauge Digitizer v.4.1 software to extract the OS and RFS data from the survival curves, which could cause a potential source of bias and have an effect on the reliability of the conclusions.

In conclusion, the present meta-analysis comparing RG and LG revealed that RG can be used safely for GC patients and provided long-term overall survival time similar to that of LG. Furthermore, large-scale and multi-center clinical RCTs are expected to assess the efficiency of RG for the treatment of GC.

## Data Availability Statement

The original contributions presented in the study are included in the article/supplementary material. Further inquiries can be directed to the corresponding author.

## Author Contributions

QF, JQ, HM, and YD contributed to the study concept and design. All authors contributed to acquisition of data. QF, JQ, and HM contributed to the analysis and interpretation of data. QF, JQ, HM, YD, PL, and GZ contributed to the drafting of the manuscript. MX contributed to the critical revision of the manuscript for important intellectual content and contributed to administrative, technical, or material support and study supervision. All authors contributed to the article and approved the submitted version.

## Funding

This work was supported by grants from the Medical and Health Science and Technology Development Research Center of the National Health Commission of China (WA2020RW12).

## Conflict of Interest

The authors declare that the research was conducted in the absence of any commercial or financial relationships that could be construed as a potential conflict of interest.

## Publisher’s Note

All claims expressed in this article are solely those of the authors and do not necessarily represent those of their affiliated organizations, or those of the publisher, the editors and the reviewers. Any product that may be evaluated in this article, or claim that may be made by its manufacturer, is not guaranteed or endorsed by the publisher.
